# A robust, highly stretchable supramolecular polymer conductive hydrogel with self-healability and thermo-processability

**DOI:** 10.1038/srep41566

**Published:** 2017-01-30

**Authors:** Qian Wu, Junjie Wei, Bing Xu, Xinhua Liu, Hongbo Wang, Wei Wang, Qigang Wang, Wenguang Liu

**Affiliations:** 1 School of Materials Science and Engineering, Tianjin Key Laboratory of Composite and Functional Materials, Tianjin University, Tianjin, 300350, China; 2 School of Chemical Science and Engineering, Tongji University, Shanghai, 200092, China

## Abstract

Dual amide hydrogen bond crosslinked and strengthened high strength supramolecular polymer conductive hydrogels were fabricated by simply *in situ* doping poly (N-acryloyl glycinamide-co-2-acrylamide-2-methylpropanesulfonic) (PNAGA-PAMPS) hydrogels with PEDOT/PSS. The nonswellable conductive hydrogels in PBS demonstrated high mechanical performances—0.22–0.58 MPa tensile strength, 1.02–7.62 MPa compressive strength, and 817–1709% breaking strain. The doping of PEDOT/PSS could significantly improve the specific conductivities of the hydrogels. Cyclic heating and cooling could lead to reversible sol-gel transition and self-healability due to the dynamic breakup and reconstruction of hydrogen bonds. The mending hydrogels recovered not only the mechanical properties, but also conductivities very well. These supramolecular conductive hydrogels could be designed into arbitrary shapes with 3D printing technique, and further, printable electrode can be obtained by blending activated charcoal powder with PNAGA-PAMPS/PEDOT/PSS hydrogel under melting state. The fabricated supercapacitor via the conducting hydrogel electrodes possessed high capacitive performances. These cytocompatible conductive hydrogels have a great potential to be used as electro-active and electrical biomaterials.

Conductive hydrogels (CHs) represent a class of functional materials, which combine the soft-wet feature of hydrogels with the electrical properties of conductive polymers^1^. CHs have found a myriad of applications, including supercapacitors, fuel cells, rechargeable lithium batteries, chemical/biosensors and biomedical devices^2^,^3^,^4^,^5^,^6^,^7^,^8^. However, conductive hydrogels are still mechanically weak and brittle, which severely hinders their practical applications^9^,^10^.

Recently, much effort has been devoted to developing high strength conductive hydrogels, including composite and double network conductive hydrogels^11^,^12^,^13^,^14^,^15^,^16^. Composite conductive hydrogels refer to the incorporation of conductive nanomaterials within hydrogel networks, such as supramolecular nanofibers^11^, carbon nanotubes^12^, and graphene oxide^13^. Double network conductive hydrogels are normally synthesized by oxidative polymerization of conductive monomers inside the double network gel matrices which are fabricated by introducing a second flexible network into the first rigid network^14^,^15^,^16^. Although these two types of conductive hydrogels are mechanically strong, their preparations involve relatively complicated processes^17^. On the other hand, the majority of conductive polymers have a low solubility in solvents, thus giving rise to processing difficulties^18^, and the heterogeneous and discontinuous distribution of conductive components in the hydrogel matrices^14^.

In recent years, self-healing properties of hydrogels have aroused increasing attention due to their promising capability of extending service life and preventing fatal failure^19^,^20^. Although self-healable and highly stretchable conductive hydrogels have been prepared^19^,^20^,^21^, balancing self-healing efficiency and high mechanical properties is still challenging. Lately, we reported on high strength thermoplastic supramolecular polymer hydrogels formed by multiple hydrogen bonding crosslinks from side chain glycinamide of monomer N-acryloyl glycinamide (NAGA)^22^. The PNAGA hydrogels demonstrated high strength, thermoplasticity and self-healability. Intriguingly, heating and cooling contributed to excellent mending of damage hydrogels. As a consequence, self-healed PNAGA hydrogels almost recover the initial mechanical strength due to the reversible breakup and reconstruction of hydrogen bonds under the condition of heating and cooling process. More importantly, the thermoplasticity will be able to allow for very facile fabrication of multiform shapes and patterns by 3D printing through thermomelting extrusion.

In this study, we aim to develop a simple but robust approach to construct a novel conductive supramolecular hydrogels with high strength, self-healability, and thermoplasticity. PNAGA serves not only as the backbone of conductive hydrogels, but also the crosslinker of copolymers. In order to tune the water absorption ability and conductivity, 2-acrylamide-2-methylpropanesulfonic acid (AMPS) was copolymerized with NAGA to form supramolecular copolymer hydrogels. We anticipate that copolymerization with hydrophilic AMPS could modulate the thermoplasticity to 3D print the hydrogels more readily. In preparing conductive hydrogels, aqueous dispersion of poly (3,4-ethylenedioxythiophene)-poly (styrenesulfonate) (PEDOT/PSS) was directly mixed with NAGA and/or AMPS monomers. The PEDOT/PSS colloidal particles are negatively charged, aiding in the stability in the aqueous media^23^. PEDOT/PSS has received wide attention, owing to its multiple merits including water-dispersibility, good conductivity, prominent chemical (environmental) stability, and excellent biocompatibility^24^,^25^. Therefore, from the perspective of preparation methods, it is facile and convenient to disperse PEDOT/PSS in the mixture uniformly and homogenously. We envision that our conductive supramolecular hydrogels with sophisticated functions may find potential applications in the fields of biosensors, electrode materials, stretchable supercapacitors, and soft tissue engineering scaffolds.

## Results

### Characterization of the conductive hydrogels

In our previous work^22^, we have demonstrated the hydrogen bonding crosslinks of dual amides in side chain could contribute to the gelation of concentrated aqueous solutions of poly(N-acryloyl glycinamide) (PNAGA). It is natural to think that copolymerization of NAGA with other hydrophilic monomer in aqueous solution may allow for facile preparation of high strength supramolecular copolymer hydrogels because of crosslinkage from PNAGA segments.

**Figure 1:**
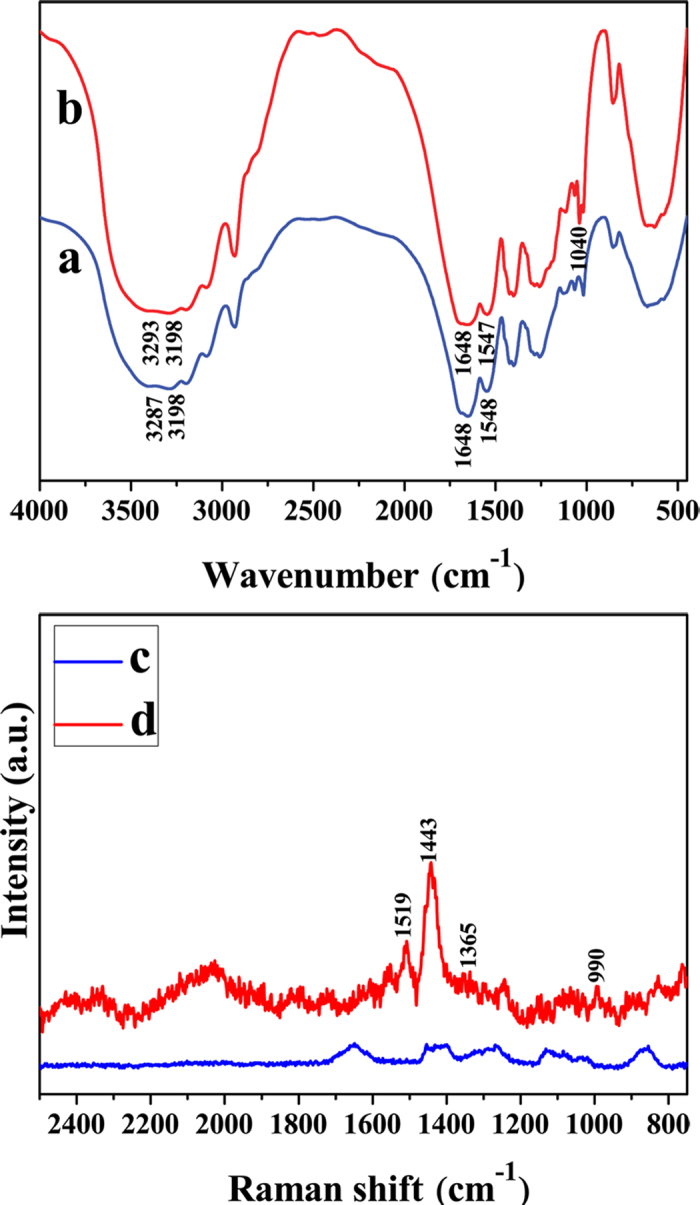
AIR-FTIR and Raman spectra of the hydrogels. FTIR spectra of PNAGA/PEDOT/PSS-0 hydrogel (a) and PNAGA-PAMPS/PEDOT/PSS-0-24 hydrogel (b). Raman spectra of PNAGA-PAMPS/PEDOT/PSS-0-24 hydrogel (c) and PNAGA-PAMPS/PEDOT/PSS-5-24 hydrogel (d).

**Figure 2:**
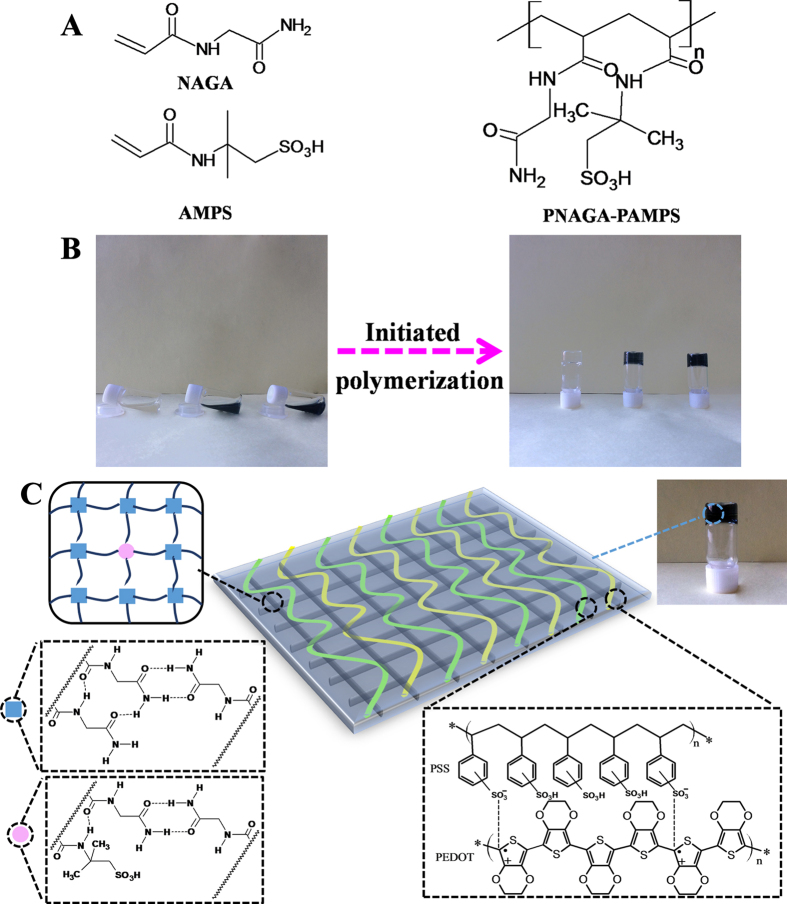
**(A)** Schematic illustration of the molecular structures of N-acryloyl glycinamide (NAGA), 2-acrylamide-2-methylpropanesulfonic acid (AMPS), and poly (NAGA-co-AMPS) (PNAGA-PAMPS). **(B)** Gelation of aqueous solutions of PNAGA-PAMPS/PEDOT/PSS-X-49 (X = 0, 3, 5 from left to right in turn) **(C)** Schematic description of the network structures crosslinked by dual amide hydrogen bonds and doped with PEDOT/PSS.

Figure 1 shows the ATR-FTIR and Raman spectra of the hydrogels. The characteristic peak at 3287 cm^−1^ is assigned to the asymmetric stretching vibrations of -NH_2_ and symmetric stretching vibrations of -NH-22,26. The band at 3198 cm^−1^ is attributed to the symmetric stretching vibrations of -NH_2_^27^. The strong absorptions at 1648 cm^−1^ and 1548 cm^−1^ arise from the vibrations of C=O and the bending vibration of -NH-, respectively^22^. The peak of acryloyl double bond of NAGA monomer at 1614 cm^−1^ is invisible in the spectrum of PNAGA, evidencing the completion of polymerization reaction^28^, and the formation of supramolecular hydrogels (Fig. 1a). Comparatively, in Fig. 1b, the feature peaks of -SO_2_- appear at 1040 cm^−1^ 27. Besides, the characteristic peaks of PNAGA are also present, suggesting the formation of PNAGA-PAMPS copolymer. ^1^H NMR specra also demonstates the formation of copolymers. Figure S2A shows ^1^H NMR spectra (D_2_O): δ = 1.8–2.1 (H_a_, polymer backbone, *-CH*_*2*_*-*), 2.4–2.6 (H_b_, polymer backbone, *-CH-*), 4.1–4.4 (H_c_, *-NH-CH*_*2*_*-CONH*_*2*_) ppm^26^. The apparent difference between Figure S2B and A is the appearance of chemical shift around at 3.3 ppm, which is due to the *-CH*_*2*_*-* in AMPS^27^. The molecular structures of NAGA, AMPS and PNAGA-PAMPS are depicted in Fig. 2A.

In Raman spectra as shown in Fig. 1c, the band at 990 cm^−1^ is assigned to oxyethylene ring deformation of PEDOT/PSS, while the broad absorption bands at 1365 cm^−1^and 1519 cm^−1^ are related to the asymmetric stretching deformation of C_α_-C_α_ and C_β_-C_β_, respectively^14^,^29^,^30^. The peak at 1443 cm^−1^ is ascribed to the symmetric stretching mode of the aromatic C_α_=C_β_ in the PEDOT^14^,^29^,^30^. In comparison, the intensities of the above bands in PNAGA-PAMPS/PEDOT/PSS-0-24 hydrogels are rather weaker compared with those of PNAGA-PAMPS/PEDOT/PSS-5-24 containing PEDOT/PSS (Fig. 1d). All these manifest that PEDOT/PSS has indeed been doped in hydrogel matrices.

In our experiment, we found 20 wt% aqueous solutions of PAMPS-PNAGA and PNAGA-PAMPS/PEDOT/PSS-X-49 could form stable supramolecular polymer hydrogels due to multiple hydrogen bonding crosslinks, as portrayed in Fig. 2B (X = 0, 3, 5 from left to right in turn) and Fig. 2C. Clearly, PNAGA-PAMPS hydrogel become dark blue with doping PEDOT/PSS whose chemical structure is delineated in Fig. 2C, suggesting that conductive components are firmly bound to the network. No leaking of PEDOT/PSS was observed even if the gels were immersed in water for a long time. Furthermore, with an increment in the concentration of PEDOT/PSS, more of conductive components are incorporated in the hydrogels, as demonstrated by darkening of color (Figure S3).

The SEM images of the free-dried hydrogels reveal that the PEDOT/PSS particles were evenly dispersed in the gel matrix (Figure S4).

### Mechanical properties

**Figure 3:**
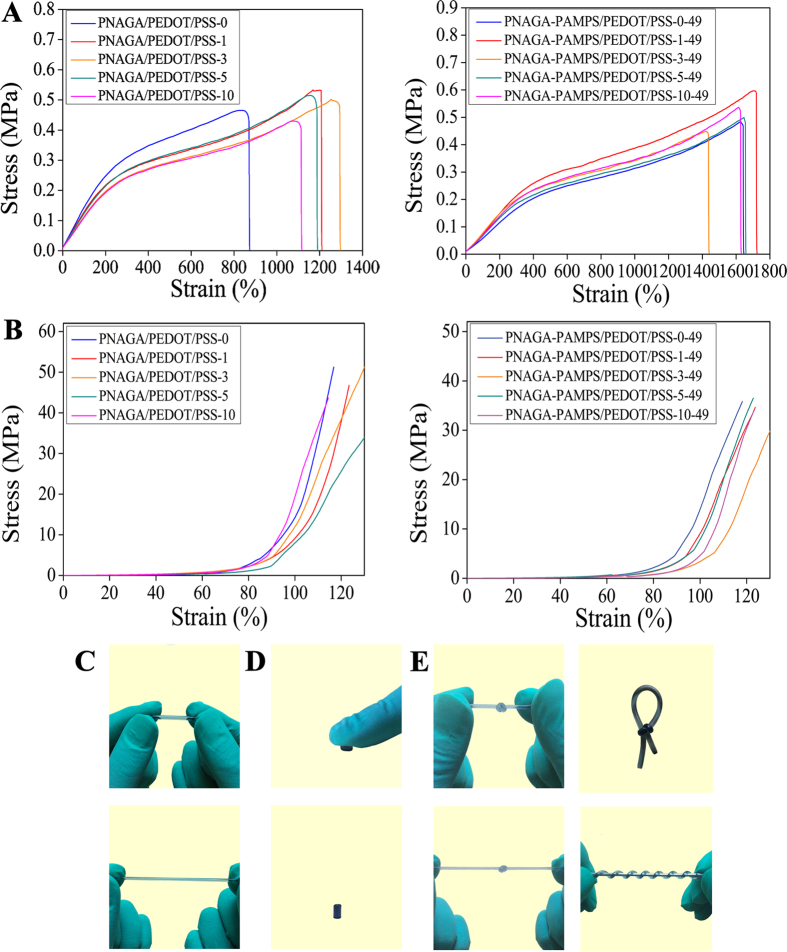
Tensile stress-strain curves of PNAGA/PEDOT/PSS-X and PNAGA-PAMPS/PEDOT/PSS-X-49 hydrogels. (**A**) Compressive stress-strain curves of PNAGA/PEDOT/PSS-X and PNAGA-PAMPS/PEDOT/PSS-X-49 hydrogels (**B**) Photographs of PNAGA-PAMPS/PEDOT/PSS-3-49 showing the ability to withstand large stretching (**C**) compression (**D**) and knotting/twisting (**E**).

The above results demonstrate that the concentrated aqueous solutions of PNAGA-PAMPS and PNAGA-PAMPS/PEDOT/PSS were still able to form supramolecular hydrogels as PNAGA homopolymer owing to the robust crosslinkage of dual amide hydrogen bonds. Next, it is essential to investigate the mechanical strengths of copolymer hydrogels. We noted that the formed supramolecular hydrogels were soaked into PBS (pH = 7.4) to reach swelling equilibrium prior to mechanical test. The equilibrium water contents (EWCs) were measured to be in the range of 65.11–87.70%, and the as-prepared hydrogels were very stable in PBS with only slight swelling. As shown in Table S2, the mechanical properties of all the hydrogels are strongly dependent on the NAGA/AMPS ratio. However, the content of PEDOT/PSS exerts a less effect on the mechanical performances. With a decrease in NAGA/AMPS ratio, the mechanical strengths of the hydrogels decline. In contrast, the mechanical properties decrease to a less degree with increment of PEDOT/PSS content. An explanation is that the decrease in NAGA ratio leads to an evident reduction in the density of hydrogen bond crosslinking. While blending PEDOT/PSS may only result in a slight disruption of hydrogen bonds. From Table S2, we can find that the hydrogels exhibit 0.22–0.58 MPa tensile strength, 1.02–7.62 MPa compressive strength, 817–1709% breaking strain, and 30–110 kPa Young’s modulus. The representative tensile/compressive stress-strain curves clearly reflect the excellent mechanical properties (Fig. 3A and B). Figure 3C,D and E show that the conductive hydrogel demonstrates an excellent ability to withstand large extension, compression, knotting and twisting. Our conductive supramolecular polymer hydrogels are far more mechanically stronger than the reported supramolecular hydrogels^31^, and equivalent to the chemically crosslinked conductive hydrogels reported so far^32^. As for cyclic tensile tests, the hysteresis loops are similar from the second compression cycle on (Figure S5), indicating the excellent recovery of mechanical property of the conductive hydrogels.

### Rheological behavior and self-healability

**Figure 4:**
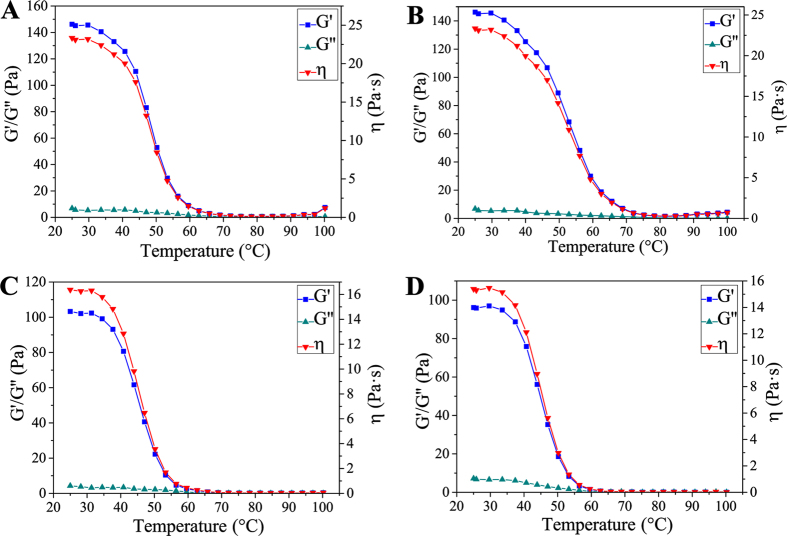
Rheological determinations of the storage modulus, loss modulus and viscosity as a function of temperature for hydrogels. **(A)** PNAGA-PAMPS-PEDOT/PSS-0-24; **(B)** PNAGA-PAMPS-PEDOT/PSS-5-24; **(C)** PNAGA-PAMPS-PEDOT/PSS-0-16; **(D)** PNAGA-PAMPS/PEDOT/PSS-5-16.

Taking into account the thermoreversibility of hydrogen bonding, we performed variable temperature rheological analysis of water swollen supramolecular hydrogels in the following experiment. Figure 4 shows that in the range of the test temperature, the higher NAGA/AMPS ratio, the greater storage modulus (G′) and loss modulus (G″) due to the denser hydrogen bonding crosslinking density. A general trend is that G′, G″ and viscosity decline with the increase of temperature. In particular, G′ and viscosity drops dramatically with temperature. Above 60 °C, G′ and G″ begin to intersect, suggesting the occurrence of the transition from gel to sol state^33^,^34^. The gel-sol transition temperatures of PNAGA-PAMPS/PEDOT/PSS-0–24, PNAGA-PAMPS/PEDOT/PSS-5-24, PNAGA-PAMPS/PEDOT/PSS-0-16 and PNAGA-PAMPS/PEDOT/PSS-5-16 are 72, 75, 65, 62 °C, respectively. Obviously, with decrease of NAGA, i.e. weakening of hydrogen bonding interaction, the evolution of gel to sol is more ready to occur; while doping PEDOT/PSS has a negligible effect on gel-sol transition. The thermoplasticity or injectability will allow for facile 3D printing of these conductive hydrogels.

**Figure 5:**
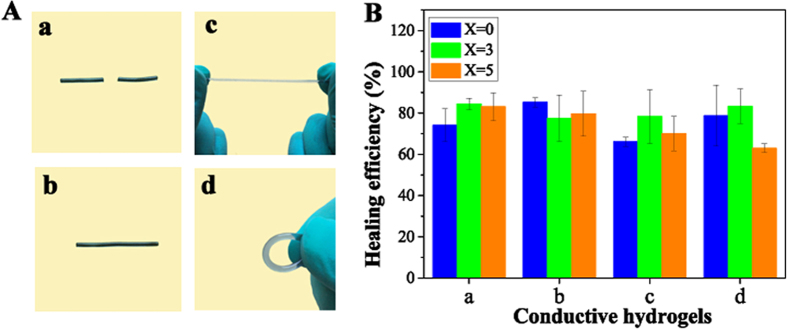
**(A)** Photographs portraying the self-healing property of the PNAGA-PAMPS-PEDOT/PSS-3-49 conductive hydrogel: (a) hydrogel was cut in the middle; (b) two halves healed completely upon heating; (c,d) the healed hydrogel can withstand stretching and bending. **(B)** Self-healing efficiency of various conductive hydrogels doped by different volume of PEDOT/PSS: (a) PNAGA/PEDOT/PSS-X hydrogels; (b) PNAGA-PAMPS/PEDOT/PSS-X-49 hydrogels; (c) PNAGA-PAMPS/PEDOT/PSS-X-24 hydrogels; (d) PNAGA-PAMPS/PEDOT/PSS-X-16 hydrogels.

The reversible breakup and reconstruction of hydrogen bonding supramolecular interactions of these conductive hydrogels suggest their self-healability. To test this ability, a representative PNAGA-PAMPS/PEDOT/PSS-3-49 hydrogel (soaked in neutral PBS) was cut into two pieces in the middle with a sharp blade. Then, the two separate surfaces were pressed together within a plastic syringe, which was immediately immersed into a 90 °C water bath for 3 h. After heat treatment, the syringe was cooled down to room temperature. Removing the syringe, the cut gel self-mended completely. The healed hydrogel can endure bending and stretching (Fig. 5A). The dynamic hydrogen bonding crosslink can allow for repeatable healing^22^. Importantly, no matter how long the cut surfaces were left, heating could re-activate the surface hydrogen bonding and eventually promote self-healing^22^. Figure 5B exhibits that the two selected hydrogels can achieve 80–85% healing efficiency. It is of note that incorporating PEDOT/PSS does not influence the self-healing properties. And the self-repaired conductive hydrogel can bear up to 500 kPa tensile strength (Figure S6).

### Conductive property

**Figure 6:**
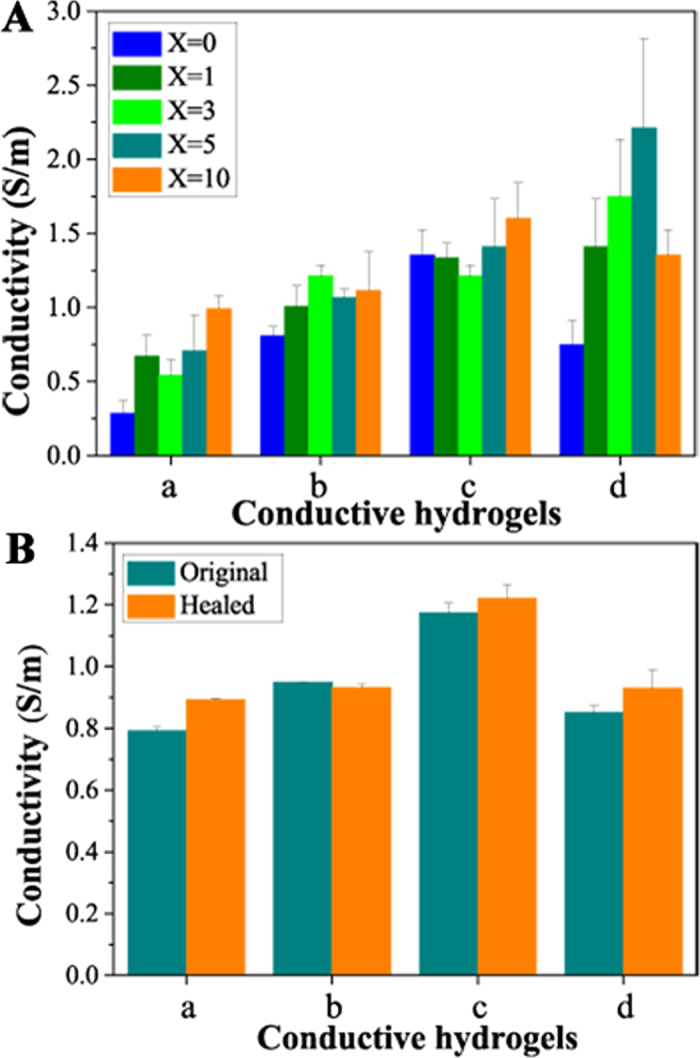
**(A)** Conductivities of hydrogels: (a) PNAGA/PEDOT/PSS-X hydrogels; (b) PNAGA-PAMPS/PEDOT/PSS-X-49 hydrogels; (c) PNAGA-PAMPS/PEDOT/PSS-X-24 hydrogels; (d) PNAGA-PAMPS/PEDOT/PSS-X-16 hydrogels. **(B)** Selected hydrogels with the fixed volume of PEDOT/PSS to evaluate the self-healing effect on the conductivities: (a) PNAGA/PEDOT/PSS-5 hydrogels; (b) PNAGA-PAMPS/PEDOT/PSS-5-49 hydrogels; (c) PNAGA-PAMPS/PEDOT/PSS-5-24 hydrogels; (d) PNAGA-PAMPS/PEDOT/PSS-5-16 hydrogels.

Figure 6A shows the conductivity of PNAGA/PEDOT/PSS-X and PNAGA-PAMPS/PEDOT/PSS-X-Y conductive hydrogels. Generally, PNAGA/PEDOT/PSS-X hydrogels become more conductive with increasing the content of PEDOT/PSS. The ionic conductivity of PNAGA/PEDOT/PSS-0 hydrogel in the absence of PEDOT/PSS is merely 0.285 S m^−1^, which is originated from the ions absorbed by hydrogel from PBS. In comparison, the remarkable increase in conductivity of PNAGA/PEDOT/PSS-X is attributed to the electrical conductivity of doped PEDOT/PSS. We can also find that the PNAGA-PAMPS hydrogels without doping PEDOT/PSS exhibit the enhanced conductivity over a certain range of AMPS. It is evident that the synergistic contribution from both ionic and electronic conduction leads to a marked enhancement in the conductivities of the PNAGA-PAMPS/PEDOT/PSS-X-Y hydrogels, and the conductivity values varies from 0.2 S m^−1^ to 2.2 S m^−1^, which are adequate to transfer bioelectrical signals *in vivo* and electrical stimulation on the cell proliferation and differentiation on account of the quite low microcurrent intensity in the human body^31^.

Next, we investigated the self-healing effects on the conductivity of hydrogels. The conductive hydrogels were cut into two pieces and healed as mentioned above. The conductivity values of heat-treated original and healed hydrogels are depicted in Fig. 6B. As shown in Fig. 6B, the conductivities of self-healing conductive hydrogels are not significantly different from those of heat-treated original hydrogels. That means that self-healing recovers not only the mechanical strengths, but also conductivities.

### 3D printing of hydrogels via thermo-processing

**Figure 7:**
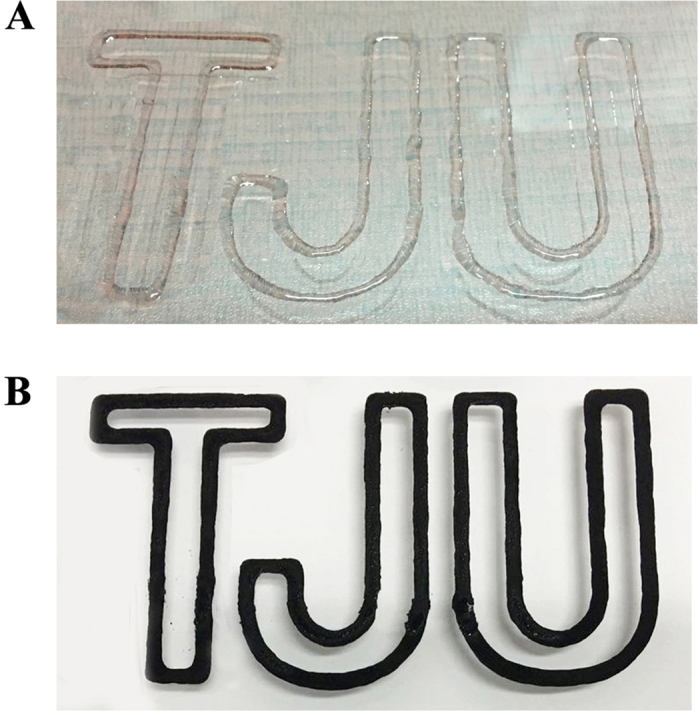
3D printing patterns of TJU with PNAGA-PAMPS/PEDOT/PSS-10-24 hydrogel (A) and PNAGA-PAMPS/PEDOT/PSS-10-24/activated charcoal (B).

In the following experiment, we investigated the feasibility of printing the supramolecular polymer hydrogels into preprogrammed shapes. Modulating the viscosity of ink is critical for the successful printing process^35^,^36^,^37^. In previous work on 3D printing of hydrogels^38^, since the viscosity of monomer was too low to maintain shape after printed, tackifier was usually added into the pregel solution. After the viscous mixture ink flowed out of nozzles, post-initiated polymerization was necessary to fix the shape. While our supramolecular polymer hydrogels excel in their reversible thermoplasticiy. They could be easily transformed into injectable liquid with appropriate viscosity by tuning temperature, and quickly solidified at room temperature to fix the shape. Herein the conductive hydrogels were fully swollen in distilled water and then heated up to transform into flow sol which was printed into preprogrammed TJU images (Acronym of Tianjin University) (Fig. 7A). At room temperature, the printed shapes could be quickly gelled due to the reformation of hydrogen bonding at low temperature.

Besides, we also blended the PNAGA-PAMPS/PEDOT/PSS-10-24 hydrogel with the activated charcoal powder to endow the hybrid hydrogel with electro-active property. The pristine hydrogel was melted at 90 °C and mixed with activated charcoal powder to form a homogenous solution, which was gelled instantly upon cooling. Then the hybrid hydrogel was mounted on the printing carriage, and heated up to 90 °C to cause a transition of sol which was extruded out of the needles readily to be printed into the predesigned TJU images (Fig. 7B). More complex structures will be able to be printed with a higher resolution 3D printer.

### Electrochemical performances

**Figure 8:**
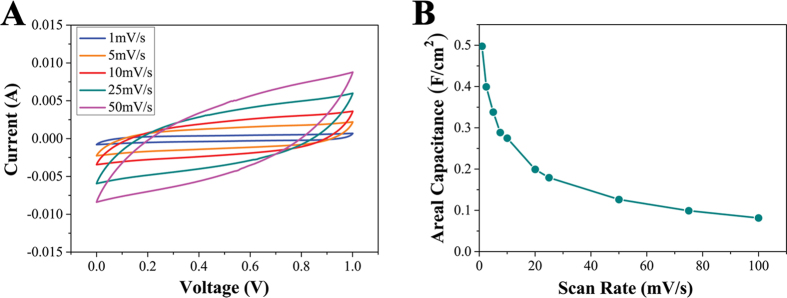
**(A)** Cyclic voltammetry curves of capacitor tested at different scan rates. **(B)** Areal capacitances of capacitor as a function of scan rates.

Next, we examined the electrochemical performances of supercapacitor fabricated by blending PNAGA-PAMPS/PEDOT/PSS-10-24 and the activated charcoal powder. As portrayed in Fig. 8A, the cyclic voltammetry curves maintain the rectangular shape at the scan rate of 25 mV s^−1^. These results indicate that the supercapacitor can endure a certain range of voltage/current change rates^21^. Although the cyclic voltammetry curves gradually deviate from the rectangular shape at a higher scan rate of 50 mV s^−1^, the loop curves of cyclic voltammetry measured at different scan rates indicate that the supercapacitor has a better electrochemical property. Figure 8B shows that the areal specific capacitance decreases with the increase of scan rates. Here areal capacitance is selected to evaluate whole hydrogel electrodes, which can represent the electrochemical performance of the real application relative to the specific capacitance divided by the mass of active carbon. It is shown that at 1 mV s^−1^ scan rate, the high areal capacitance value is up to 0.498 F cm^−2^, and at 25 mV s^−1^ scan rate, areal capacitance still remains 0.179 F cm^−2^, which is good enough (even at high scan rate, the areal capacitance is not bad). All those results demonstrate that the supercapacitor has excellent electrochemical performances. The high strength thermoplastic supramolecular polymer conductive hydrogel mixed with electroactive substances has the great potential to be printed into supercapacitors with 3D print technology.

### Cytotoxicity assay *in vitro*

Cytotoxicity is one of the critical factors determining whether a hydrogel can be used in the biomaterial field. The vitro cytotoxicity of the hydrogels was evaluated with a MTT assay. It is seen that the conductive gels can maintain 74–100% cell viability, indicating the better cytocompatibility (Figure S7).

## Discussion

In this work, we proposed a universal and robust strategy to construct high strength conductive hydrogels by simply *in situ* doping the supramolecular copolymer hydrogels crosslinked by multiple hydrogen bonds with PEDOT/PSS during polymerization. The conductive hydrogels exhibited high mechanical tensile/compressive strengths, large extensibility, and up to 2.2 S m^−1^ conductivity. Importantly, the hydrogels could seamlessly self-heal, contributing to the recovery of the mechanical properties and conductivities. The hydrogels exhibited reversible sol-gel transition due to dynamic responsiveness of hydrogen bonding. Because of thermoplasticity, the conductive could be printed into preprogrammed shapes with 3D printing technology under melting state. The cytocompatible conductive hydrogels have a great potential as biosensors, flexible electrode materials, and electroactive scaffold materials for soft tissue engineering.

## Methods

### Preparation of hydrogels

N-acryloyl glycinamide (NAGA) was synthesized by the previously described method^22^. Poly(N-acryloyl glycinamide) (PNAGA), poly(NAGA-co-AMPS) and PEDOT/PSS-doped hydrogels were prepared in terms of the formulations as shown in Table S1. Herein, the synthesis of conductive hydrogels was taken as an example. First, an appropriate quantity of monomers was dissolved in deionized water to form clear and transparent solution. All the concentrations of total monomers were set at 20 wt%. Next, a specific volume of PEDOT/PSS was added into the monomer solution and vortexed vigorously to form a homogeneous mixture. It is worth mentioning that the solution became deep blue in color at this stage. After that, 3 wt% of APS (relative to the mass of total monomers) was added into the solution and stirred thoroughly under nitrogen atmosphere, and then the solution was placed into an ice bath for 5 minutes. Subsequently, 3 wt% of TEMED (relative to the mass of total monomers) was added into the solution. After homogenization, the cooled mixture was quickly injected between two plastic rectangle molds (length 70 mm, width 50 mm) separated by a rubber gasket (thickness 0.5 mm) or plastic cylinder-shaped molds, and kept at room temperature for 24 h. Finally, the hydrogels formed were demolded and immersed into the neutral PBS solution (pH = 7.4) or deionized water to remove the impurities at room temperature. The resultant conductive hydrogels were labeled as PNAGA/PEDOT/PSS-X or PNAGA-PAMPS/PEDOT/PSS-X-Y, where X indicates the concentration of PEDOT/PSS (in % volume of the dissolved mixture) and Y represents the mass ratio of NAGA/AMPS.

### Mechanical measurement

All mechanical properties of the hydrogel samples were tested on a WDW-05 electromechanical tester (Time Group Inc, China) at room temperature. In this study, all hydrogels were immersed in PBS solution (pH = 7.4) to reach swelling equilibrium before test, and at least four specimens were tested for each measurement. For tensile strength measurements, specimens were cut into dumbbell-shaped samples in accordance to GBT 528-20094 (width: 2 mm, gauge length: 10 mm, thickness: 0.5 mm) using a customized cutter (Xuan Yu Inc, China). The rate of extension was set at 50 mm min^−1^. For compression tests, the fully swollen hydrogels were cut into cylinders (4 mm in diameter and 4 mm in height) and measured using the same tester at a fixed rate of 10 mm min^−1^. For cyclic loading-unloading tensile tests, hydrogel samples were immersed in PBS solution (pH = 7.4) to reach swelling equilibrium before test. And the four continuously repeated loading-unloading tensile cycles were measured with the fixed strain (ε = 400%) at room temperature. It is of note that all of the stresses measured are engineering stresses, which are calculated according to the following formula:


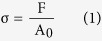


where F is the applied load and A_0_ is the starting cross-sectional area.

### Rheology measurement

Dynamic rheological experiments were performed on a rheometer (HAKKE MARS III, Germany) equipped with a plate with a diameter of 35 mm and a Peltier device for temperature control. During all rheological measurements, a solvent trap was used to minimize the evaporation. The hydrogel samples were about 1 mm in thickness and the stress σ was set at 15 Pa, which ensure that the oscillatory deformation was within the linear regime. Temperature sweep ranging from 25 to 100 °C was performed at a frequency of 1 Hz, corresponding to an angular frequency ω = 6.3 rad/s. The storage moduli (G′) and loss moduli (G″) of distinct hydrogels were recorded as a function of temperature. The hydrogel samples were fully swollen in deionized water at room temperature before test.

### Evaluation of self-healing efficiency

The original hydrogels were prepared in a glass syringe (2.0 mm in diameter, 50 mm in length). The gels were fully swollen in the PBS (pH = 7.4) before being cut into two separate pieces using a surgical knife. Then the two separated pieces were brought into contact with each other within a glass syringe. The syringe was placed into a 90 °C water bath for about 3 hours. The pristine uncut hydrogel was subjected to the same heating treatment, taking the evaporation of water into consideration. To evaluate the healing efficiency, the heat-treated and self-repaired hydrogels were soaked into neutral PBS (pH = 7.4) for 3 days, and the tensile strengths were tested. The healing efficiency (HE) is defined as follows:


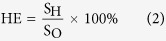


where S_H_ is the tensile strength of self-healed hydrogels, and S_O_ is the tensile strength of heated original hydrogels. All experiments were performed in triplicate for each specimen.

### Ionic conductivity measurement

The hydrogel samples (12.0 mm in diameter, 1.0 mm in thickness) were sandwiched between two stainless steel plate electrodes to form a two-electrode system. All the samples were immersed in PBS solution (pH = 7.4) at least for 3 days before test. The conductivities of different samples were determined from impedance spectra based on AC impedance method measured by a Metrohm Autolab PGSTA302N potentiostats-galvanostats (The Netherlands) coupled with a computer. And the data were treated by Zview software. The conductivities were calculated by the following equation^39^:


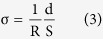


where R is the resistance; d and S are thickness and area of the specimens, respectively. For further analysis of self-healing effects on the conductivities, cylinder samples fully swollen in PBS (pH = 7.4) were cut in the middle using a surgical knife. Then, the two separated pieces were brought into contact with each other within a mold (10.0 mm in diameter, 8.0 mm in height), which was then placed into the water bath of 90 °C for 3 hours. The pristine hydrogel was also subjected to the same heating treatment, taking the evaporation of water into consideration. Finally, the conductivities of the heat-treated original and self-healed hydrogel samples were measured in the same way mentioned above after the gels were soaked in the PBS solution (pH = 7.4) for at least 3 days.

### 3D printing of conductive hydrogels

Hydrogels with specific shapes can be produced *via* 3D printing. First, the conductive hydrogel was immersed in deionized water to reach swelling equilibrium. Then, the gel was loaded into the extrusion carriages of the 3D printer (Nano-Plotter NP 2.1, GeSiM, Grosserkmannsdorf, Germany). Further, in order to avoid the blockage of the needles, the hydrogel was converted into the high viscosity solution by heating up to 90 °C and then cooling to 60 °C, by which the solution could be extruded from the needles at the speed of 10 mm s^−1^ by printing machine. The size of needle diameter is 1 mm. Upon existing, the printed shapes were quickly fixed at room temperature due to the reconstruction of hydrogen bonding.

Similarly, conductive hydrogel blended with activated charcoal powder (YP 80F, 2100 m^2^ g^−1^) was also printed. The activated charcoal powder with 0.8–1.35 nm pore sizes was blended into the hydrogel under melting state prior to printing. The content of the activated charcoal powder was fixed at 10 wt% of the hydrogel. Then the mixture was loaded into the extrusion carriages of the 3D printer, and extruded out of the needles with the same process. The printed shapes solidfied rapidly at room temperature.

### Fabrication of supercapacitors

The mixture of hydrogels and the activated charcoal powder was used to fabricate supercapacitors and the electrochemical properties of the supercapacitors were measured by the method of cyclic voltammetry. The preparation of supercapacitors mainly comprised the following steps. First, the selected hydrogel samples, which reached swelling equilibrium in deionized water, were heated up to 90 °C to realize the gel-sol transition. Next, the activated charcoal powder was added into the sol. After being stirred and vibrated, the uniform and flow mixture of activated charcoal powder and sol was spread on the two copper foil current collectors. The content of the activated charcoal powder was 10 wt% of the whole hydrogel. Then, the mixture was loaded on the two copper foil collectors and cooled down to room temperature. After that, the cellulose membrane was placed between two electrodes, with 0.6 M bistrifluoromethanesulfonimide lithium salt (LiTFSi) being used as the electrolyte. The electroactive properties of the obtained supercapacitors were evaluated by the method of cyclic voltammetry, which was carried out by a Metrohm Autolab PGSTA302N potentiostats-galvanostats (The Netherlands) coupled with a computer. In order to analyze the variation of capacitance applying various scan rates, the areal capacitance was calculated by the following equation based the curve of cyclic voltammetry^40^.


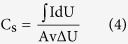


where C_s_ indicates the areal capacitance; ∫ IdU the integrated area of the curve of cyclic voltammetry; I is the current; A is the surface area of active materials in electrodes; v is the scanning speed and ΔU is the voltage window.

## Additional Information

**How to cite this article**: Wu, Q. *et al*. A robust, highly stretchable supramolecular polymer conductive hydrogel with self-healability and thermo-processability. *Sci. Rep.* **7**, 41566; doi: 10.1038/srep41566 (2017).

**Publisher's note:** Springer Nature remains neutral with regard to jurisdictional claims in published maps and institutional affiliations.

## Supplementary Material

Supplementary Information
