# Supplementary material for: A robust, highly stretchable supramolecular polymer conductive hydrogel with self-healability and thermo-processability
Source: Sci Rep. 2017 Jan 30;7:41566. doi: 10.1038/srep41566 (PMC5278500; doi:10.1038/srep41566)
Supplement: Supplementary Information [file srep41566-s1.doc]

**Supplementary Information**

**A robust, highly stretchable supramolecular polymer conductive hydrogel with self-healability and thermo-processability**

Qian Wu^1^, Junjie Wei^2^, Bing Xu^1^, Xinhua Liu^2^, Hongbo Wang^1^, Wei Wang^1^, Qigang Wang^2,*^, and Wenguang Liu^1,*^

^1^School of Materials Science and Engineering, Tianjin Key Laboratory of Composite and Functional Materials, Tianjin University, Tianjin, 300350, China;

*E-mail: [wgliu@tju.edu.cn](mailto:wgliu@tju.edu.cn)

^2^School of Chemical Science and Engineering, Tongji University, Shanghai, 200092, China;

*E-mail: wangqg66@tongji.edu.cn

**Materials.** Glycinamide hydrochloride (purity >98%) and acryloyl chloride (purity >98%) were purchased from Tokyo Kasei Kogyo Company in Japan. Poly(3,4-ethylenedioxythiophene)-poly(styrenesulfonate) (PEDOT/PSS, 1.3 wt % dispersion in H_2_O) was supplied by Tianjin Heowns Biochem LLC. Ammonium persulfate (APS, purity >98%) was obtained from Aladdin Biochemical Polytron Technologies Inc in Shanghai. [2-Acrylamide-2-methylpropanesulfonic acid](http://www.lookchem.com/2-Acrylamide-2-methylpropanesulfonic-acid/) (AMPS, 98%) and *N, N, N′, N′*-tetramethylethylenediamine (TEMED, 99%) were purchased from Alfa Aesar Chemical Co., Ltd in Tianjin. Activated charcoal powder (YP 80F, 2100 m^2^ g^-1^) was purchased from Kuraray Co., Ltd. All other chemicals and solvents were of analytical grade.

**Measurement of molecular weight.** The molecular weight and polydispersity index (PDI) were measured by GPC on a waters Model TDA 302 instrument, using 0.1mol L^-1^ NaNO_3_ solution as the eluent at a flow rate of 1.0 mL min^-1^. Polyethylene oxide standard was used for calibration. The number average molecular weight (M_n_) of PNAGA-PAMPS/PEDOT/PSS was determined to be 170,000, and its polydispersity index (PDI) was 1.1 (Fig. S1, ESI†).

**ATR-FTIR and Raman spectroscopy.** Attenuated total reflection Fourier transform infrared (ATR-FTIR) spectroscopy conducted on a Perkin Elmer spectrum 100 (USA) was applied to confirm the successful the formation of the hydrogels.

Raman spectroscopy was used to recognize the existence of conductive components. The spectra were recorded on a ThermoFisher DXR Raman Microscope with an excitation wavelength of 532 nm in dynamic mode from 0 to 3200 cm^−1^ at a resolution of 1 cm^−1^.

**NMR spectroscopy.** ^1^H NMR spectra of the copolymers were measured on a Varian INOVA spectrometer (500 MHz) using D_2_O as a solvent.

**Determination of equilibrium water content (EWC).** The EWCs of PNAGA/PEDOT/PSS-X and PNAGA-PAMPS/PEDOT/PSS-X-Y hydrogels fully swollen in PBS (pH = 7.4) were measured at room temperature with gravimetric method. The hydrogel samples were taken out, gently blotted with filter paper to remove water on the surface, and weighed on a microbalance. Afterwards, the hydrogel samples were dried to constant weights in a vacuum oven at 60 °C. The EWC is defined as:

$\text{EWC =}\frac{\text{m}_{\text{wet}}\text{-m}_{\text{dry}}}{\text{m}_{\text{wet}}}\times\text{100\%}$ (1)

Where m_wet_ and m_dry_ stand for the wet weight and dry weight of samples, respectively. At least four measurements were taken for each specimen.

**Field emission scanning electron microscopy (FE-SEM).** The morphologies of the hydrogel samples were observed on a field emission scanning electron microscope (SEM, FEI Quanta S-4800 FE-SEM) at accelerated electron energy of 5.0 kV. The hydrogel specimens were freeze-dried by liquid nitrogen for 5 minutes and immediately lyophilized for 48 hours. The samples were further sputter-coated with gold prior to observation.

**Cytotoxicity Tests.** The cytotoxicity of conductive hydrogels was tested using Mouse fibroblast cells (L929, obtained from Peking Union Medical College). Mouse fibroblast cells were seeded in a 48-well plate coated with hydrogels and incubated for 48 hours to evaluate the cytotoxicity of different hydrogel samples by the method of MTT assay. The culture medium was replaced with 400 μL fresh medium containing 40 μL MTT (3-(4, 5-dimethyl-2-thia-zoyl)-2, 5-diphenyl tetrazolium bromide, 5 mg/mL in PBS), and the cells were incubated for another 4 hours. Finally, the whole medium was replaced with 300 μL DMSO per well to dissolve the formed crystals and the plate was gently shaken for 15 minutes. The absorbance (Abs) of each well was measured at 490 nm on a Σ960 plate-reader (Metertech) with pure DMSO as a blank. The non-treated cells (in DMEM) were used as a control and the relative cell viability (RCV, mean% ± SD, n = 3) was expressed as below:

$RCV=\frac{Abs_{s}}{Abs_{c}}\times100\%$ (2)

Where Abs_s_ and Abs_c_ are the absorbance of the sample and control group, respectively


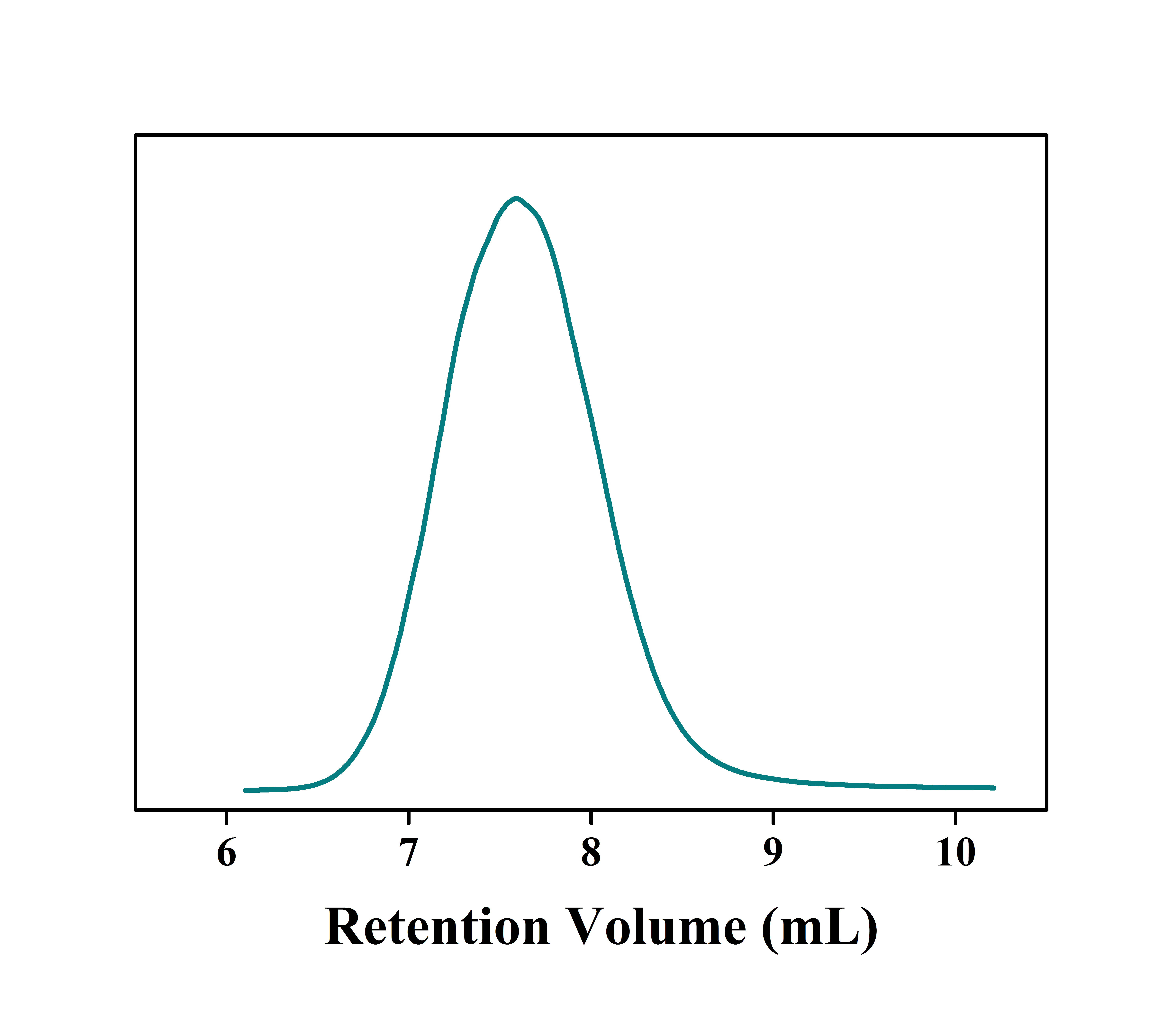


**Figure S1.** Gel permeation chromatography (GPC) elution trace of PNAGA-PAMPS/PEDOT/PSS-0-24.

**
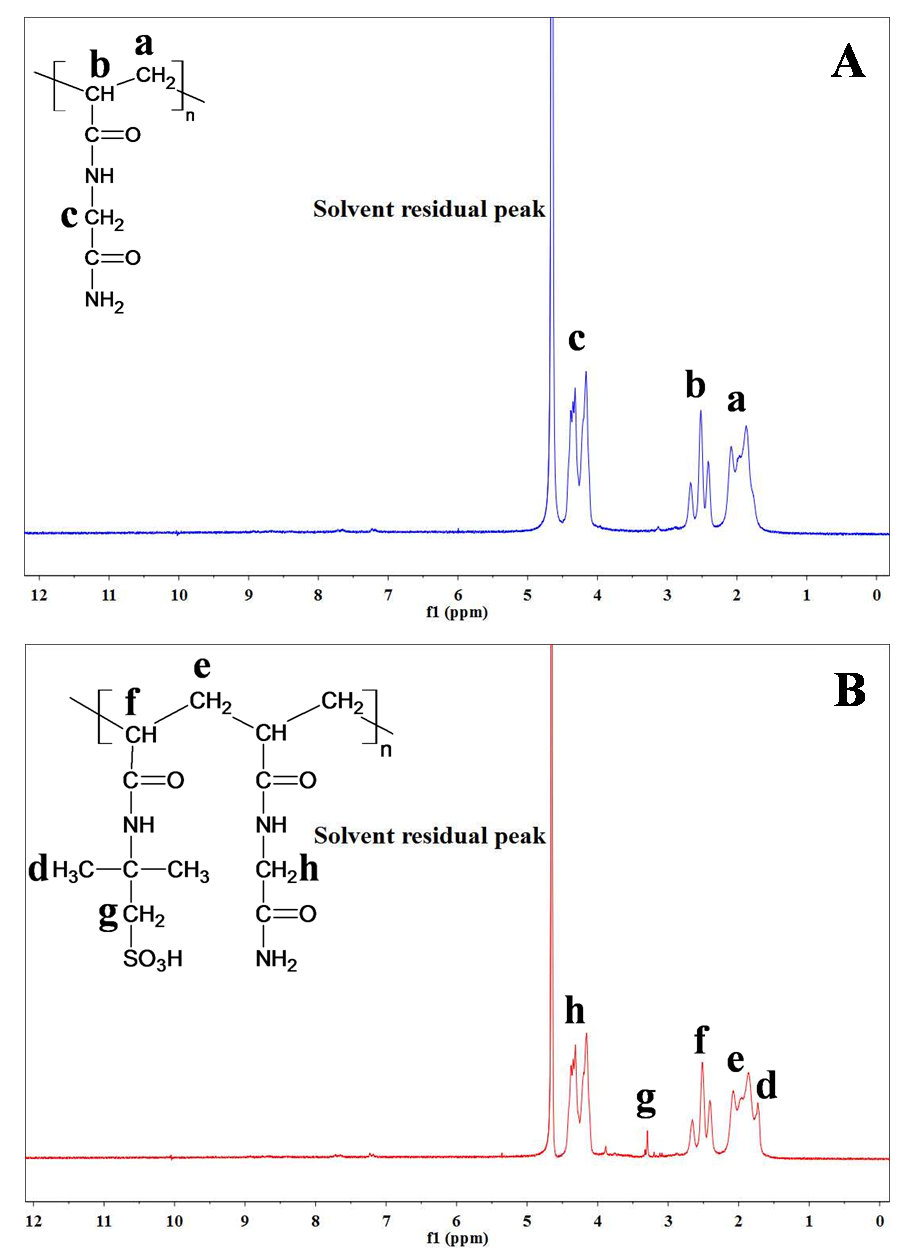
**

**Figure S2.** ^1^H NMR spectra of **(A)** PNAGA/PEDOT/PSS-0 and **(B)** PNAGA-PAMPS/PEDOT/PSS-0-24 in D_2_O.


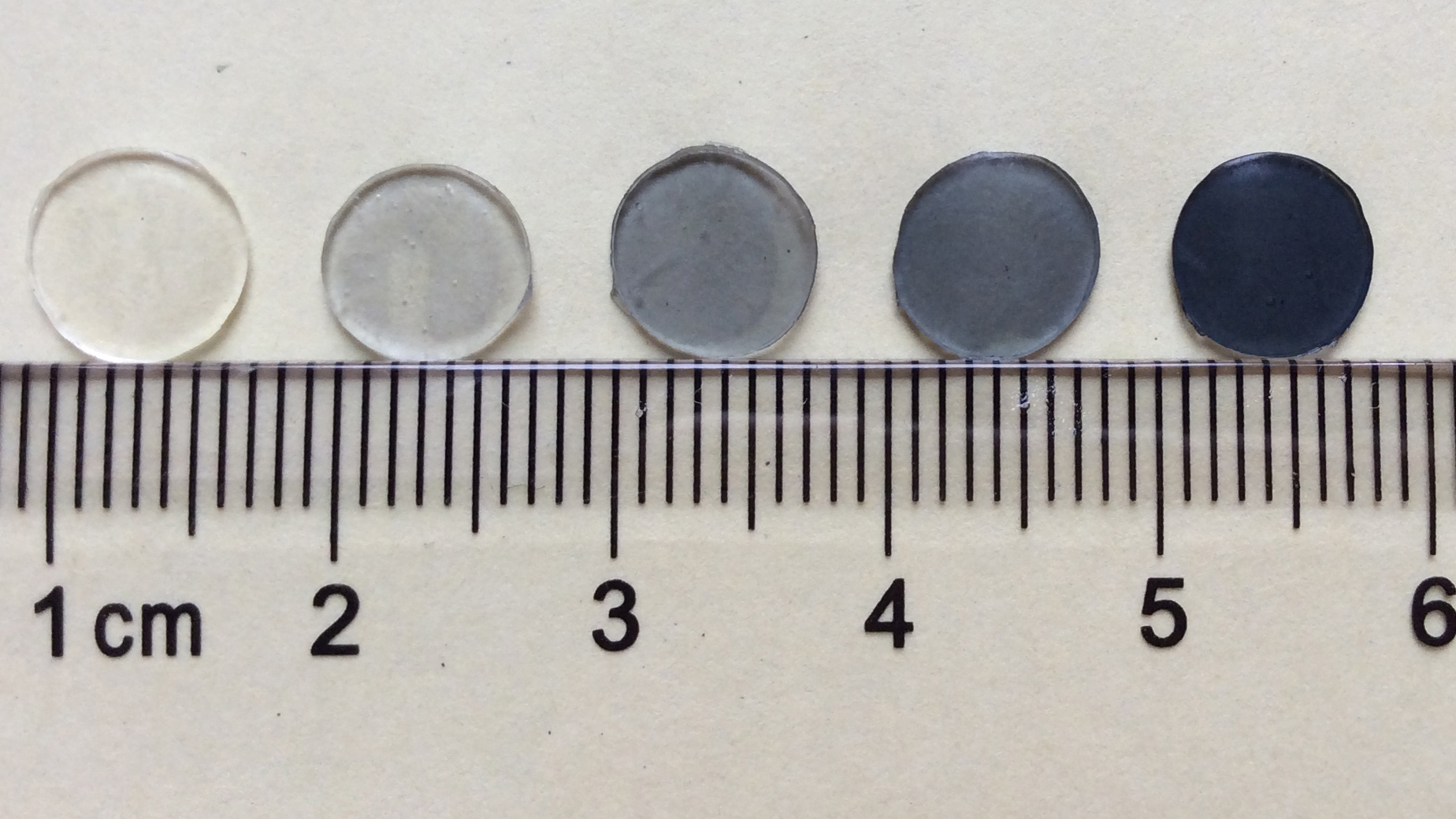


**Figure S3.** Photographs of PBS-soaked PNAGA-PAMPS/PEDOT/PSS-X-49 hydrogels (X=0, 1, 3, 5, 10 from left to right in turn) with different volumes of PEDOT/PSS.


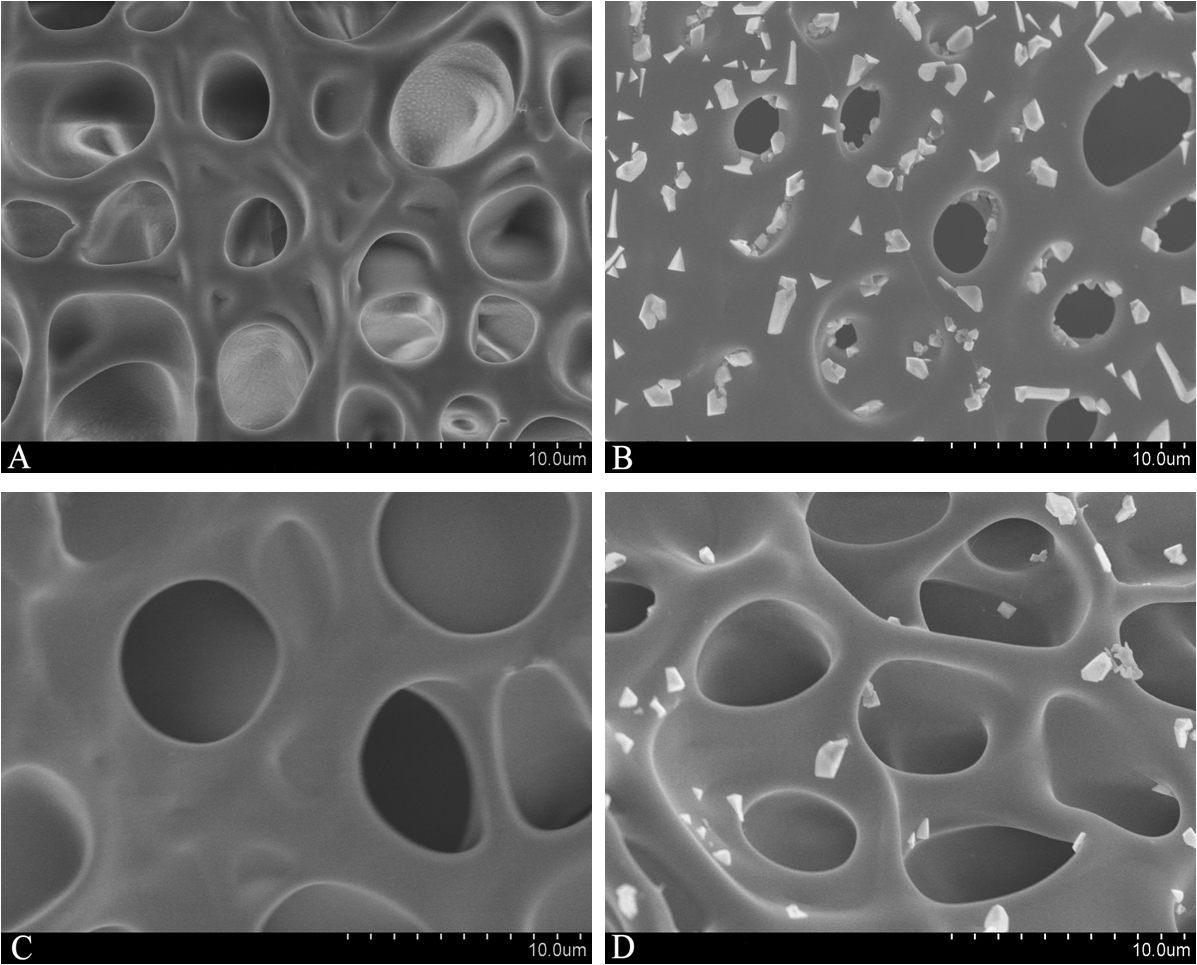


**Figure S4.** SEM images of freeze-dried hydrogels: **(A)** PNGAG-PAMPS/PEDOT/PSS-0-24 hydrogel. **(B)** PNAGA-PAMPS/PEDOT/PSS-5-24 hydrogel. **(C)** PNGAG-PAMPS/PEDOT/PSS-0-16 hydrogel. **(D)** PNAGA-PAMPS/PEDOT/PSS-5-16 hydrogel.


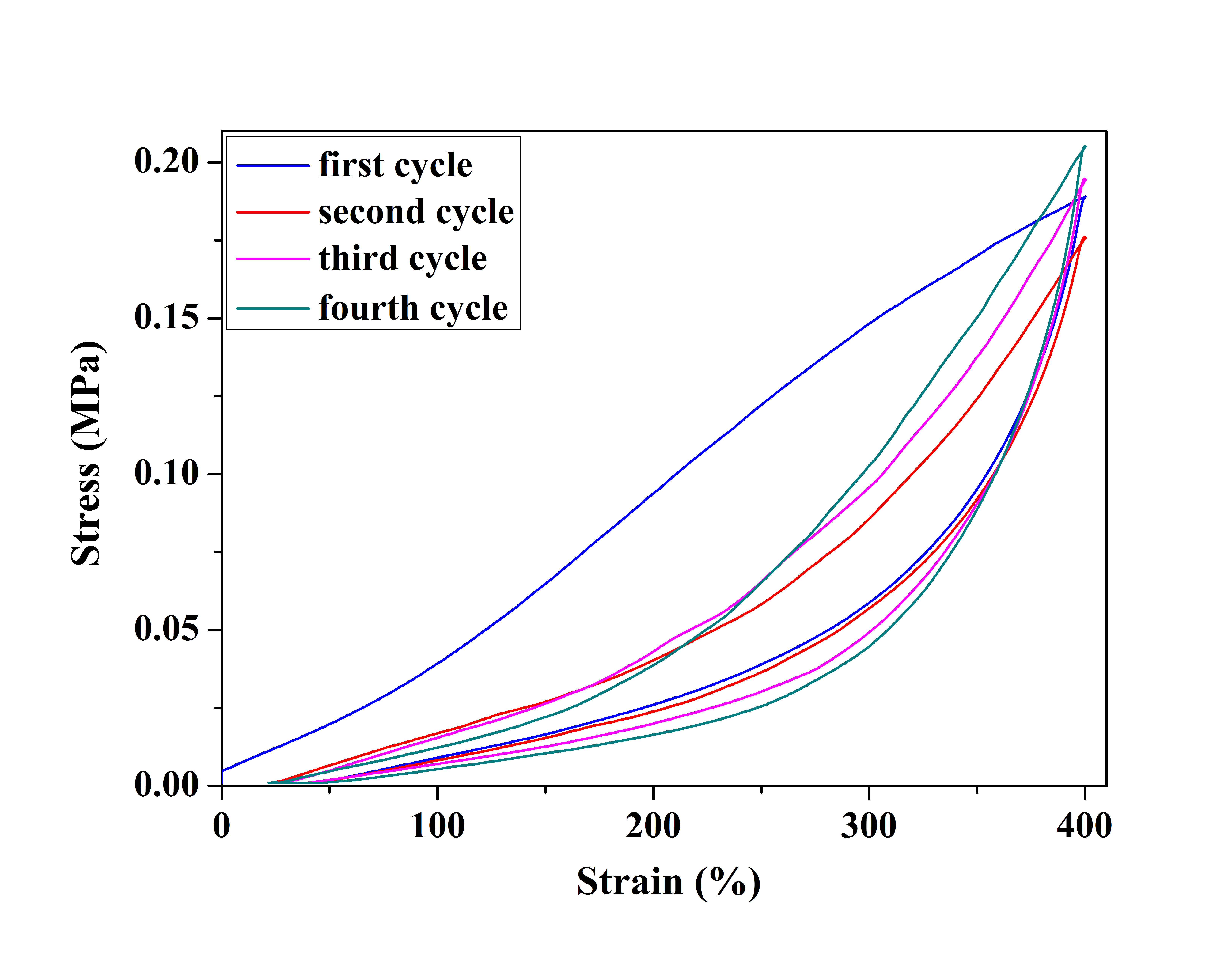

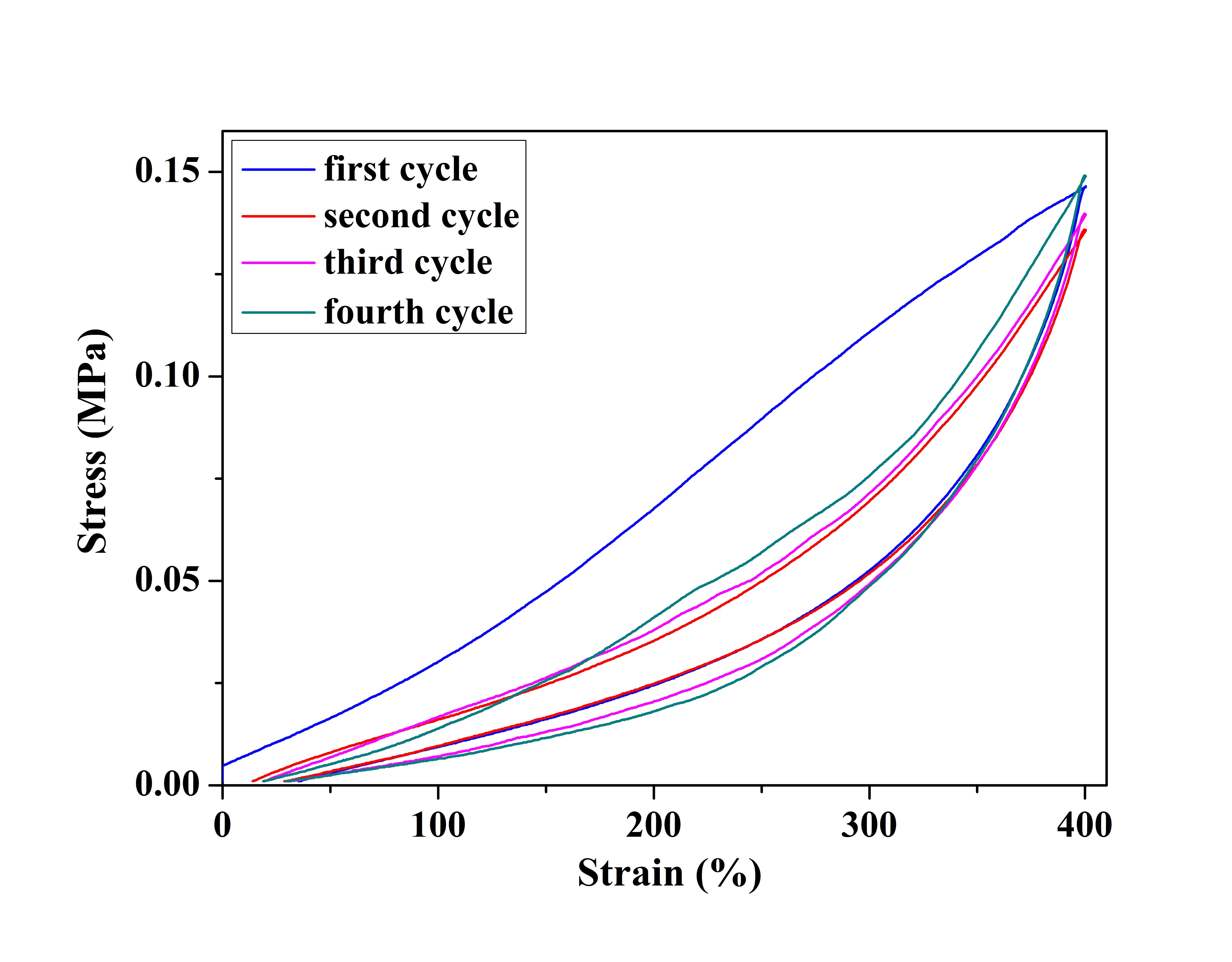

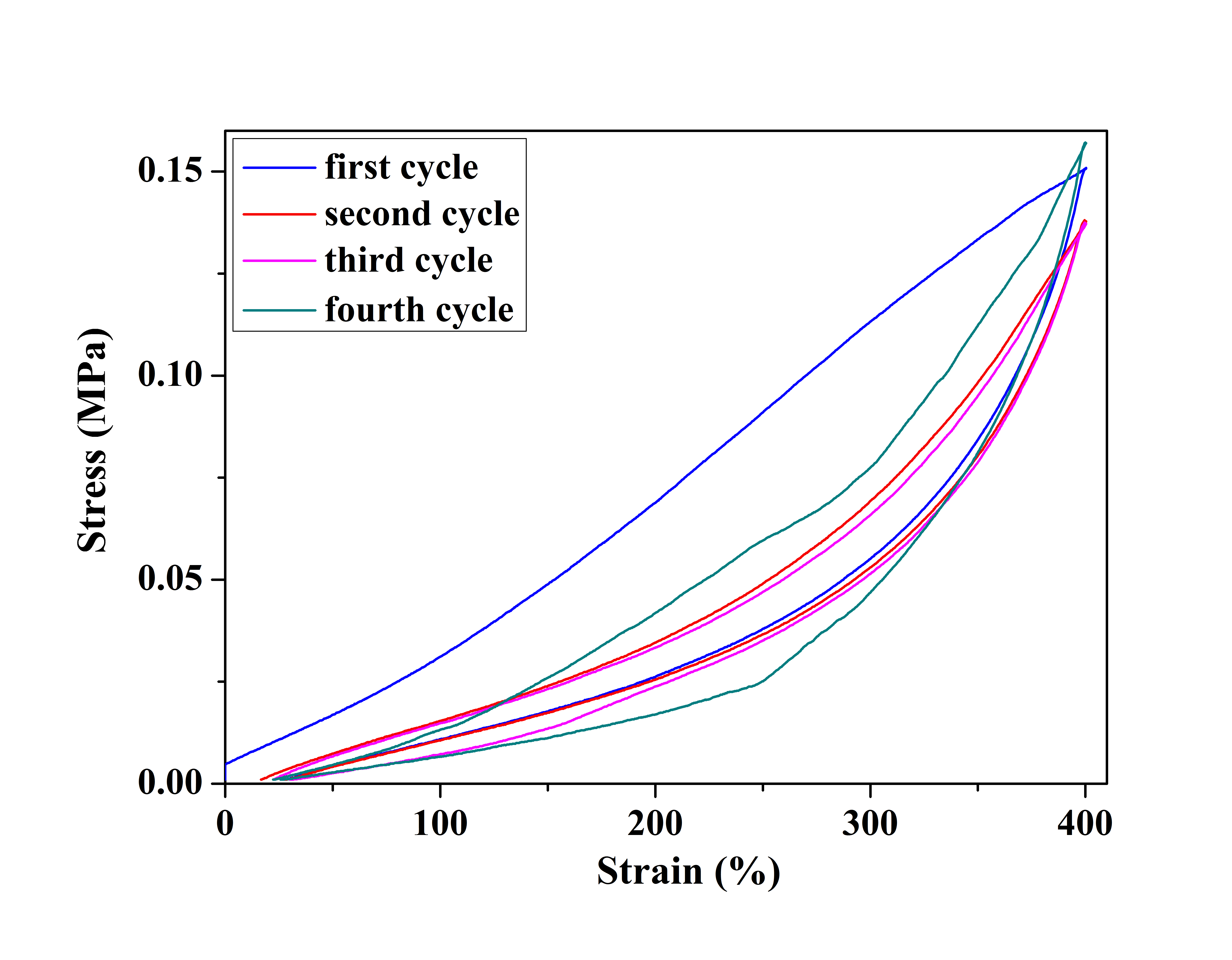


**A**

**B**

**C**

**Figure S5.** Cyclic tensile tests of conductive hydrogels: (A) PNAGA-PAMPS/PEDOT/PSS-0-24; (B) PNAGA-AMPS/PEDOT/PSS-5-24; (C) PNAGA-PAMPS/PEDOT/PSS-10-24.


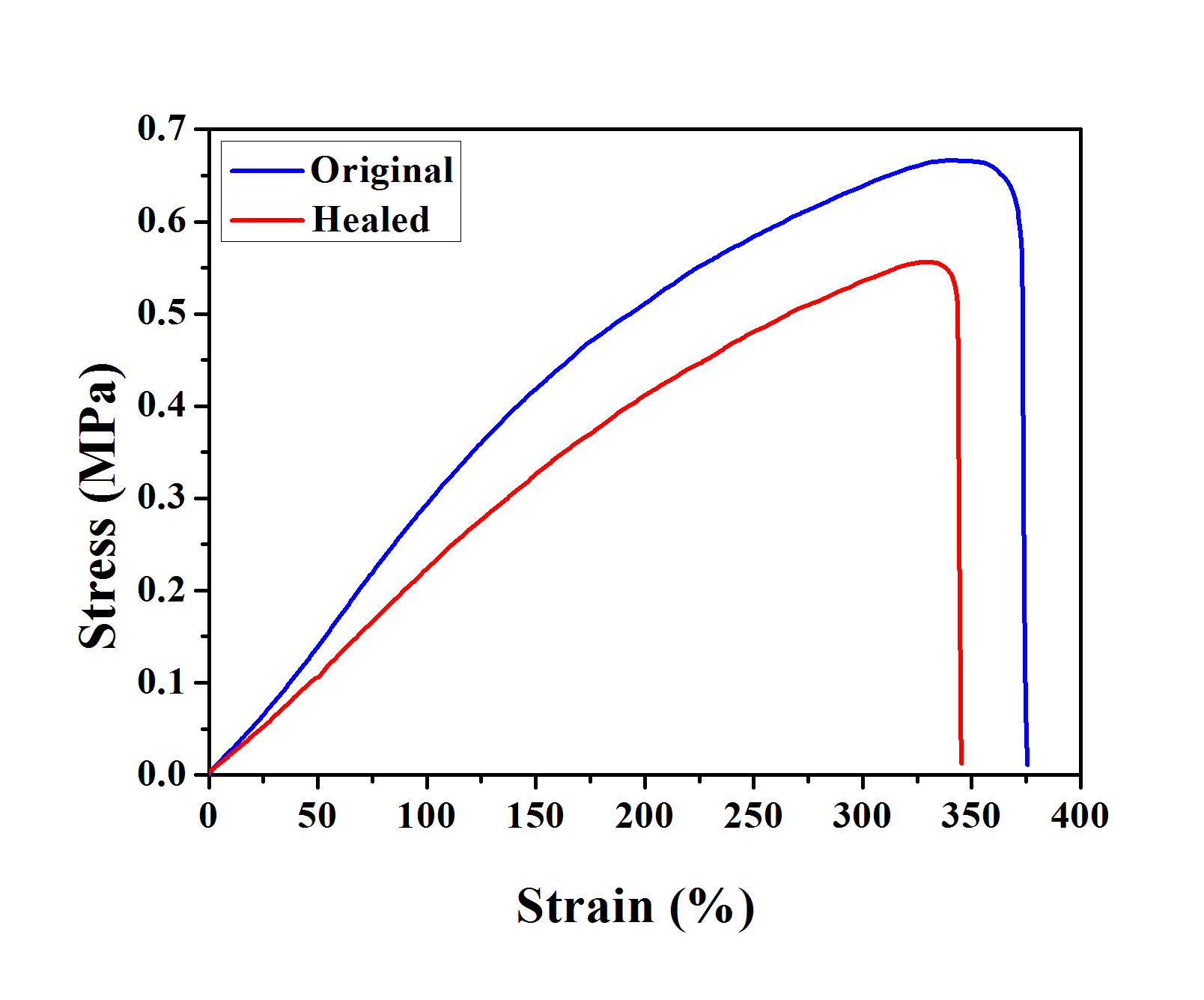


**A**


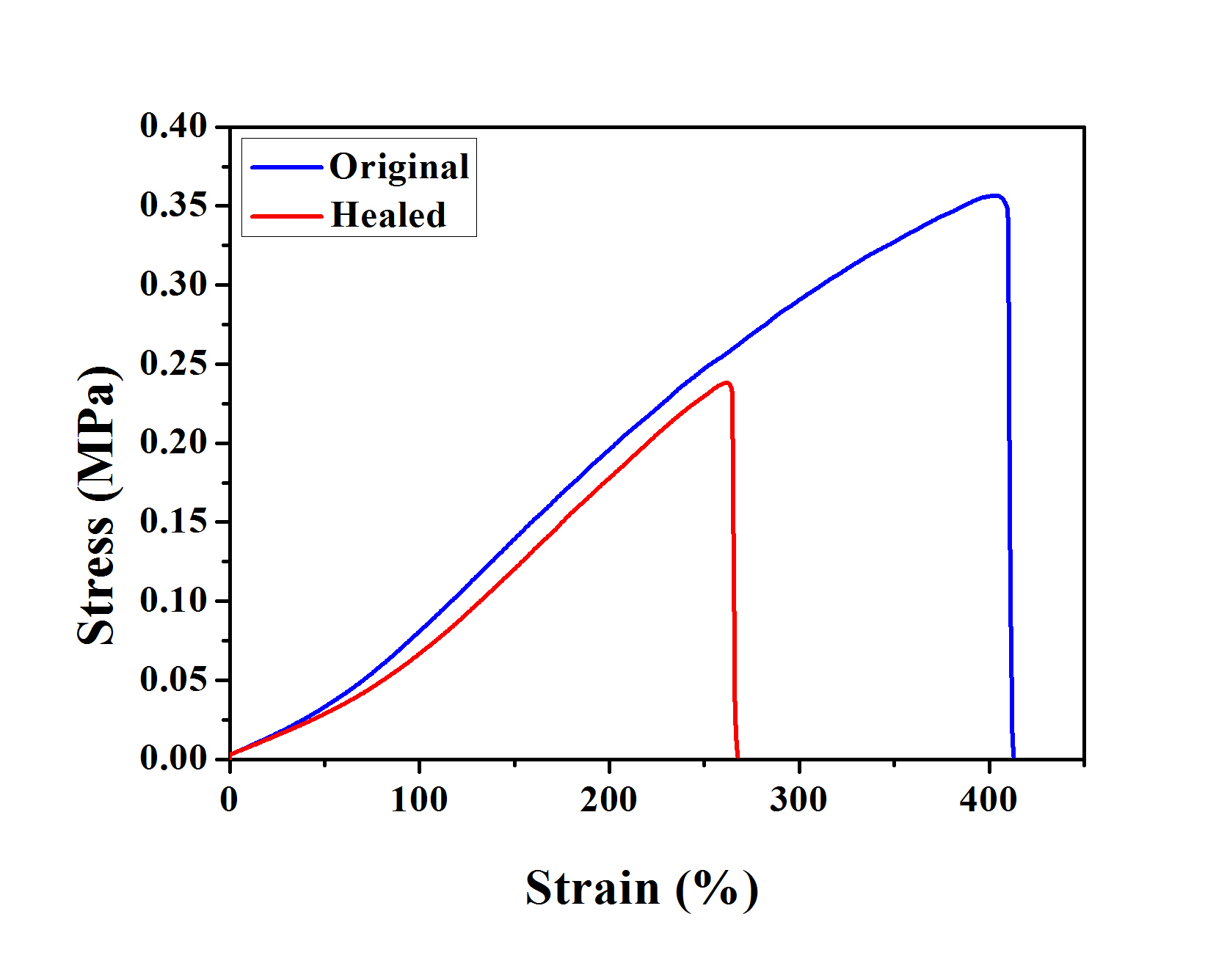


**B**

**Figure S6.** (**A).** Tensile stress-strain curves of heat-treated original and healed PNAGA/PEDOT/PSS-5 hydrogel. **(B)**. Tensile stress-strain curves of heat-treated original and healed PNAGA-PAMPS/PEDOT/PSS-5-24 hydrogel.


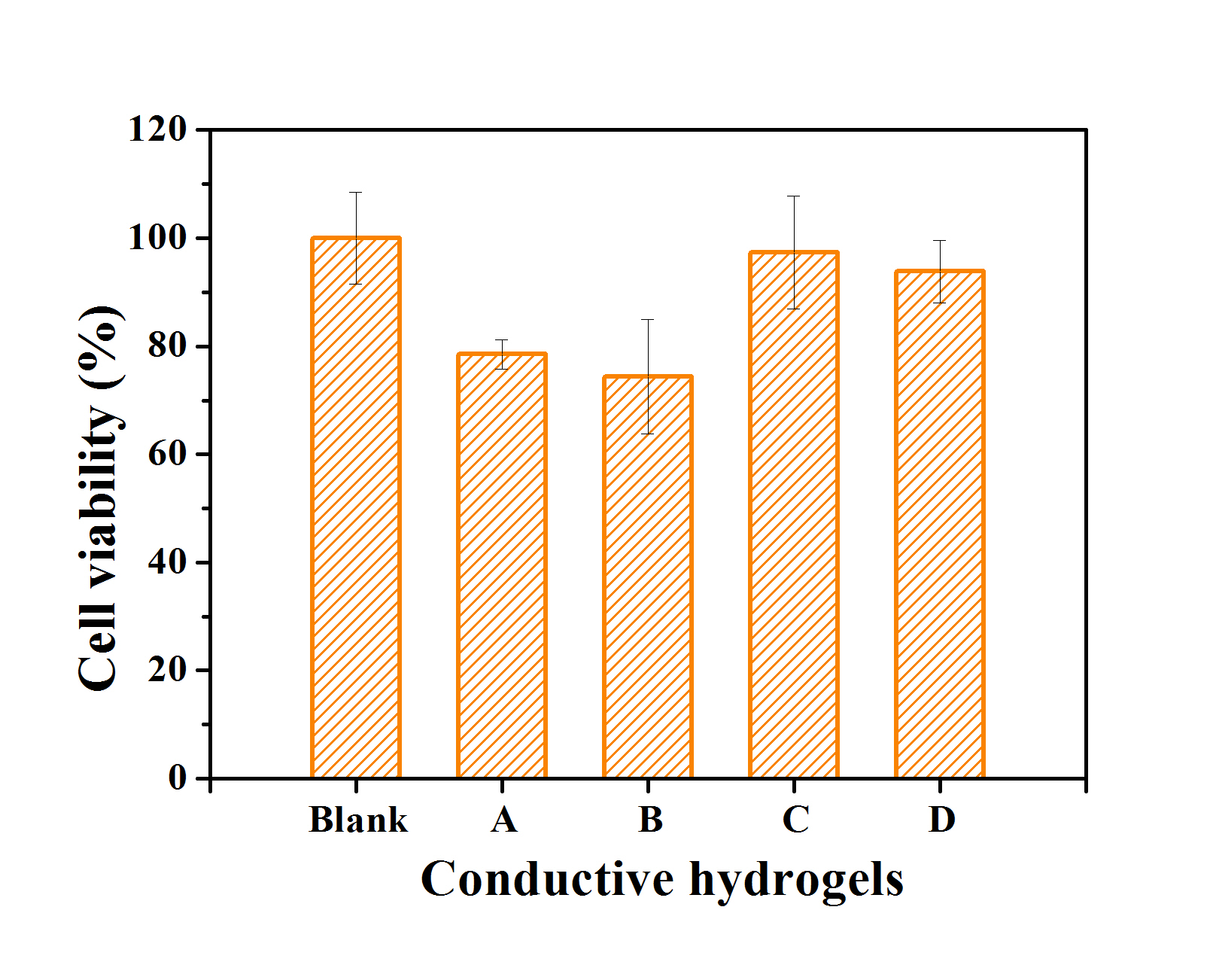


**Figure S7.** Cytotoxicity results (represented by percentage cell viability) of different conductive hydrogel samples: **(A)** PNAGA/PEDOT/PSS-0 hydrogel; **(B)** PNAGA/PEDOT/PSS-5 hydrogel; **(C)** PNAGA-PAMPS/PEDOT/PSS-0-24 hydrogel; **(D)** PNAGA-PAMPS/PEDOT/PSS-5-24 hydrogel. Blank is the cells seeded on culture plate.

**Table S1.** Varied formulations for preparing conductive hydrogels doped with different volumes of PEDOT/PSS.

| Sample | NAGA (mg) | AMPS (mg) | PEDOT/PSS (μL) |
| --- | --- | --- | --- |
| PNAGA/PEDOT/PSS-0 | 100 | 0 | 0 |
| PNAGA/PEDOT/PSS-1 | 100 | 0 | 5 |
| PNAGA/PEDOT/PSS-3 | 100 | 0 | 15 |
| PNAGA/PEDOT/PSS-5 | 100 | 0 | 25 |
| PNAGA/PEDOT/PSS-10 | 100 | 0 | 50 |
| PNAGA-PAMPS/PEDOT/PSS-0-49 | 98 | 2 | 0 |
| PNAGA-PAMPS/PEDOT/PSS-1-49 | 98 | 2 | 5 |
| PNAGA-PAMPS/PEDOT/PSS-3-49 | 98 | 2 | 15 |
| PNAGA-PAMPS/PEDOT/PSS-5-49 | 98 | 2 | 25 |
| PNAGA-PAMPS/PEDOT/PSS-10-49 | 98 | 2 | 50 |
| PNAGA-PAMPS/PEDOT/PSS-0-24 | 96 | 4 | 0 |
| PNAGA-PAMPS/PEDOT/PSS-1-24 | 96 | 4 | 5 |
| PNAGA-PAMPS/PEDOT/PSS-3-24 | 96 | 4 | 15 |
| PNAGA-PAMPS/PEDOT/PSS-5-24 | 96 | 4 | 25 |
| PNAGA-PAMPS/PEDOT/PSS-10-24 | 96 | 4 | 50 |
| PNAGA-PAMPS/PEDOT/PSS-0-16 | 94 | 6 | 0 |
| PNAGA-PAMPS/PEDOT/PSS-1-16 | 94 | 6 | 5 |
| PNAGA-PAMPS/PEDOT/PSS-3-16 | 94 | 6 | 15 |
| PNAGA-PAMPS/PEDOT/PSS-5-16 | 94 | 6 | 25 |
| PNAGA-PAMPS/PEDOT/PSS-10-16 | 94 | 6 | 50 |

**Table S2.** Mechanical properties and EWCs of conductive hydrogels prepared with different formulations

| Samples | Tensile strength (MPa) | Elongation at break (%) | Young’s modulus (MPa) | Compressive strength at 90% strain (MPa) | Compressive modulus (MPa) | EWC (%) |
| --- | --- | --- | --- | --- | --- | --- |
| PNAGA/PEDOT/PSS-0 | 0.54±0.063 | 817.6±49.0 | 0.11±0.010 | 7.62±3.48 | 0.61±0.14 | 66.74±1.0 |
| PNAGA/PEDOT/PSS-1 | 0.57±0.027 | 1210.3±40.8 | 0.067±0.010 | 4.08±0.45 | 0.52±0.073 | 66.68±1.6 |
| PNAGA/PEDOT/PSS-3 | 0.50±0.020 | 1235.3±19.9 | 0.077±0.011 | 4.92±0.86 | 0.59±0.12 | 65.11±0.80 |
| PNAGA/PEDOT/PSS-5 | 0.51±0.011 | 1156.5±80.5 | 0.080±0.010 | 2.92±1.46 | 0.38±0.18 | 65.95±0.50 |
| PNAGA/PEDOT/PSS-10 | 0.45±0.044 | 1074.4±32.7 | 0.083±0.012 | 6.93±3.90 | 0.58±0.053 | 65.77±0.60 |
| PNAGA-PAMPS/PEDOT/PSS-0-49 | 0.49±0.038 | 1646.1±29.1 | 0.033±0.010 | 5.30±2.40 | 0.36±0.14 | 70.89±1.0 |
| PNAGA-PAMPS/PEDOT/PSS-1-49 | 0.58±0.015 | 1673.4±88.2 | 0.060±0.022 | 3.12±0.48 | 0.32±0.056 | 71.46±2.1 |
| PNAGA-PAMPS/PEDOT/PSS-3-49 | 0.46±0.022 | 1458.4±99.1 | 0.050±0.010 | 2.24±0.81 | 0.29±0.020 | 72.01±0.60 |
| PNAGA-PAMPS/PEDOT/PSS-5-49 | 0.49±0.021 | 1619.9±57.7 | 0.040±0.010 | 3.26±0.80 | 0.35±0.022 | 71.97±0.60 |
| PNAGA-PAMPS/PEDOT/PSS-10-49 | 0.53±0.011 | 1619.9±16.5 | 0.043±0.011 | 2.31±0.48 | 0.25±0.037 | 73.60±0.70 |
| PNAGA-PAMPS/PEDOT/PSS-0-24 | 0.33±0.059 | 1352.2±54.7 | 0.043±0.012 | 1.43±0.16 | 0.095±0.025 | 77.22±0.90 |
| PNAGA-PAMPS/PEDOT/PSS-1-24 | 0.50±0.020 | 1709.7±63.1 | 0.033±0.010 | 1.67±0.33 | 0.066±0.040 | 82.54±0.70 |
| PNAGA-PAMPS/PEDOT/PSS-3-24 | 0.41±0.022 | 1616.1±13.8 | 0.033±0.011 | 1.56±0.11 | 0.12±0.032 | 78.59±0.50 |
| PNAGA-PAMPS/PEDOT/PSS-5-24 | 0.34±0.070 | 1476.4±16.6 | 0.033±0.010 | 1.68±0.10 | 0.089±0.032 | 78.00±0.60 |
| PNAGA-PAMPS/PEDOT/PSS-10-24 | 0.35±0.020 | 1589.5±16.5 | 0.030±0.011 | 1.02±0.16 | 0.082±0.025 | 79.10±0.50 |
| PNAGA-PAMPS/PEDOT/PSS-0-16 | 0.24±0.016 | 844.1±61.5 | 0.040±0.012 | 2.86±1.17 | 0.065±0.030 | 86.65±1.0 |
| PNAGA-PAMPS/PEDOT/PSS-1-16 | 0.24±0.090 | 846.4±29.7 | 0.043±0.013 | 2.33±0.51 | 0.054±0.016 | 86.71±0.10 |
| PNAGA-PAMPS/PEDOT/PSS-3-16 | 0.21±0.050 | 841.0±29.6 | 0.040±0.010 | 4.00±0.41 | 0.072±0.010 | 87.06±0.10 |
| PNAGA-PAMPS/PEDOT/PSS-5-16 | 0.22±0.010 | 1023.4±24.6 | 0.030±0.011 | 3.04±0.82 | 0.067±0.010 | 87.06±0.10 |
| PNAGA-PAMPS/PEDOT/PSS-10-16 | 0.23±0.050 | 1161.9±86.8 | 0.030±0.010 | 4.73±1.40 | 0.089±0.034 | 87.70±0.10 |
